# Neurological Impairment in Critically Ill Patients on Dialysis: Research Letter for the INCOGNITO-AKI Feasibility Study

**DOI:** 10.1177/20543581231192743

**Published:** 2023-08-24

**Authors:** Natasha A. Jawa, Samuel A. Silver, Rachel M. Holden, Stephen H. Scott, Andrew G. Day, Patrick A. Norman, Benjamin Y. M. Kwan, David M. Maslove, John Muscedere, J. Gordon Boyd

**Affiliations:** 1 Centre for Neuroscience Studies, School of Medicine, Queen’s Health Sciences, Queen’s University, Kingston, ON, Canada; 2 Division of Nephrology, Department of Medicine, Queen’s University, Kingston, ON, Canada; 3 Department of Biomedical and Molecular Sciences, Queen’s University, Kingston, ON, Canada; 4 Kingston General Health Research Institute, Kingston, ON, Canada; 5 Department of Public Health Sciences, Queen’s University, Kingston, ON, Canada; 6 Department of Diagnostic Radiology, Queen’s University, Kingston, ON, Canada; 7 Department of Critical Care Medicine, Queen’s University, Kingston, ON, Canada; 8 Department of Critical Care Medicine, Kingston Health Sciences Centre, Kingston, ON, Canada; 9 Division of Neurology, Department of Medicine, Queen’s University, Kingston, ON, Canada

**Keywords:** cerebral oxygenation, neurocognitive impairment, kidney replacement therapy, neuroimaging, critical illness

## Abstract

**Background:**

Acute kidney injury (AKI) resulting in kidney replacement therapy is rising among critically ill adults. Long-term kidney replacement therapy and critical illness are independently linked to acute and prolonged cognitive impairment, and structural brain pathology. Poor regional cerebral oxygenation (rSO_2_) may be a contributing factor.

**Objective:**

To assess the feasibility of testing the association between intradialytic rSO_2_ and acute and long-term neurological outcomes.

**Design:**

Longitudinal observational study.

**Setting and Participants:**

We enrolled patients initiating continuous kidney replacement therapy or intermittent hemodialysis in the Kingston Health Sciences Centre (KHSC) Intensive Care Unit (ICU).

**Measurements and Methods:**

rSO_2_ was monitored during the first 72 hours of continuous kidney replacement therapy or throughout each intermittent hemodialysis session. We measured acute neurological impairment by daily delirium screening and long-term neurocognitive outcomes using the Kinarm robot, Repeatable Battery for the Assessment of Neuropsychological Status, and brain magnetic resonance imaging.

**Results:**

Of 484 ICU patients, 26 met the screening criteria. Two declined, and 13 met at least one exclusion criteria. Eleven patients were enrolled. Eight died in ICU, one died 2 months after discharge, and one declined follow-up. Data capture rates were high: rSO_2_/vitals (91.3%), and delirium screening and demographics (100%). Longitudinal testing was completed in 50% (1 of 2) of survivors.

**Limitations:**

Enrollment was low due to a variety of factors, limiting our ability to evaluate long-term outcomes.

**Conclusion:**

rSO_2_ and delirium data collection is feasible in critically ill patients undergoing kidney replacement therapy; high mortality limits follow-up.

## Abrégé

**Contexte:**

L’insuffisance rénale aiguë (IRA) menant à une thérapie de remplacement rénal est en augmentation chez les adultes aux soins intensifs. Un séjour aux soins intensifs et la thérapie de remplacement rénal à long terme sont indépendamment liés à des déficits cognitifs aigus et prolongés ainsi qu’à des pathologies structurelles du cerveau. La faible saturation régionale du cerveau en oxygène (rSO2) pourrait être un facteur contributif.

**Objectif:**

Évaluer la possibilité de tester l’association entre la rSO_2_ intradialytique et les résultats neurologiques aigus et chroniques.

**Type d’étude:**

Étude observationnelle longitudinale.

**Cadre et sujets de l’étude:**

Nous avons recruté des patients qui entamaient une thérapie de remplacement rénal en continu ou une hémodialyse intermittente à l’unité des soins intensifs (USI) du Kingston Health Sciences Centre (KHSC).

**Mesures et méthodologie:**

La rSO2 a été surveillée pendant les 72 premières heures de thérapie de remplacement rénal en continu, ou tout au long de chaque séance d’hémodialyse intermittente. Nous avons mesuré les déficits neurologiques aigus par un dépistage quotidien du délirium et les atteintes neurocognitives à long terme à l’aide du robot Kinarm, de la *Repeatable Battery for the Assessment of Neuropsychological Status* et de l’imagerie par résonance magnétique cérébrale.

**Résultats:**

Sur les 484 patients hospitalisés à l’USI, 26 répondaient aux critères de sélection. Deux ont refusé de participer à l’étude et treize satisfaisaient à au moins un critère d’exclusion. Onze patients ont été inclus à l’étude. Huit patients sont décédés à l’USI, un est décédé deux mois après sa sortie de l’hôpital et un a refusé le suivi. Les taux de saisie des données étaient élevés: rSO2 et paramètres vitaux (91,3 %), dépistage du délirium et démographie (100 %). Des tests longitudinaux ont été effectués chez 50 % (1 de 2) des survivants.

**Limites:**

Le taux d’inscription était faible en raison de divers facteurs, ce qui a limité notre capacité à évaluer les résultats à long terme.

**Conclusion:**

Il est possible de collecter des données sur la rSO2 et le délirium chez les patients de soins intensifs qui suivent une thérapie de remplacement rénal; un taux de mortalité élevé a limité le suivi.

**Trial Registration:**

clinicaltrials.gov, registration number NCT04722939

## What was known before

- Long-term kidney replacement therapy (KRT) and critical illness are independently linked to acute and prolonged cognitive impairment, and structural brain pathology.
- Low regional cerebral oxygen saturation (rSO_2_) may be related to both acute and long-term neurological impairments in this vulnerable cohort of patients; long-term follow-up of these patients is needed to further understand this relationship.
- No studies to date have explored the feasibility of longitudinal multimodal data capture in this critically ill cohort of patients on dialysis.

## What this adds

- This study sought to determine whether undertaking a longitudinal follow-up study of critically ill patients initiated on dialysis in the intensive care unit (ICU) was feasible in our single center, as a first step toward being able to understand the neurological issues facing this cohort, and ultimately develop targeted interventions to improve neurological complications in this population.
- It is feasible to collect regional cerebral oxygenation and delirium data in critically ill patients undergoing kidney replacement therapy. Long-term follow-up may be challenging in this cohort due to high mortality.

## Introduction

The prevalence of acute kidney injury (AKI) is increasing among critically ill patients admitted to the intensive care unit (ICU), with over half of adult ICU patients meeting the criteria for AKI at some point during their admission, and up to 13% receiving treatment with kidney replacement therapy (KRT).^1^ Although KRT may be a lifesaving intervention for individuals with kidney failure, *long-term (ie, maintenance)* KRT is associated with poor neurocognitive outcomes and reduced quality of life.^2^ Critical illness has also independently been linked to prolonged cognitive impairment and structural brain pathology.^3^ Therefore, adults on *short-term* KRT for AKI in the ICU may be at risk for superimposed impairments associated with both critical illness and dialysis-requiring AKI. Unfortunately, the neurocognitive and structural neurological impact of KRT in this population remains largely unknown.

Delirium is one of the most consistent and reliable risk factors for cognitive impairment among critically ill adults^4^ and has been associated with increased brain atrophy.^3^ Delirium is common among ICU patients, particularly in those with AKI.^5^ Regional cerebral oxygen saturation (rSO_2_) is associated with delirium in critically ill adults,^6^ and duration of disturbed cerebral autoregulation is correlated with the duration of delirium.^7^ Therefore, rSO_2_ may provide an early marker of neurocognitive impairment.

The rationale for this program of research is the lack of data examining the association between KRT, delirium, and long-term structural and cognitive outcomes in critically ill patients. Prior to embarking on the full study, this feasibility study was conducted to assess participant enrollment rates, data fidelity, and long-term follow-up. We identified barriers to enrollment or data collection to design mitigation strategies to ensure adherence to a complex protocol involving longitudinal multimodal data collection.

## Methods

This study was approved by the Queen’s University Health Sciences and Affiliated Teaching Hospitals Research Ethics Board (DMED-2424-20). The methodological details for the full study have been published.^8^ Briefly, participants were eligible for inclusion if they met the following criteria: age ≥18 years; diagnosis of severe AKI Stage 2/3 requiring KRT (per KDIGO criteria); and within 12 hours of initiation of KRT via intermittent hemodialysis (IHD) or continuous kidney replacement therapy (CKRT). Exclusion criteria were as follows: acquired/congenital neurological disorders (impacting neurological function independent of kidney disease/KRT); contraindication to testing with cerebral oximetry, Kinarm, or magnetic resonance imaging (MRI)^30^); KRT via peritoneal dialysis (uncommon in the critical care setting resulting in few participants for subgroup analysis); life expectancy <24 hours (impeding follow-up); clinical suspicion of renal obstruction, rapidly progressive glomerulonephritis or interstitial nephritis (which could independently affect outcomes); or prehospitalization estimated glomerular filtration rate (eGFR) <30 mL/min/1.73 m^2^ (as chronic kidney disease independently affects neurological function).

### Baseline Data Collection

Baseline illness severity was evaluated using the Acute Physiology and Chronic Health Evaluation II (APACHE II) scoring system. Baseline cognition was measured using the Clinical Dementia Rating (CDR) scale. Baseline frailty was assessed via the Clinical Frailty Scale (CFS). All assessments were performed by a trained member of the research team at the time of enrollment (N.A.J.).

### Physiological Data Collection

Participants on CKRT underwent continuous cerebral oximetry using the FORESIGHT Elite cerebral oximeter (Edwards LifeSciences, USA) during the first 72 hours of CKRT. Post-CKRT oximetry was measured for 1 hour following CKRT. Participants on IHD underwent oximetry beginning 1 hour prior to each session, continuously throughout every session, and ending 1 hour following session completion, up to a maximum of 30 days. Heart rate, peripheral oxygen saturation, blood pressure, and mean arterial pressure were continuously captured alongside cerebral oximetry. Data were extracted from the patient’s bedside vitals monitor using either BedMaster Solutions (https://www.bedmaster.net/) or MediCollector (https://www.medicollector.com/) depending on the availability for the patient.

### ICU Outcome Data Collection

The presence and severity of delirium was assessed daily using the Confusion Assessment Method (CAM)-ICU-7. Assessments were performed by a trained research team member (N.A.J.) for up to 30 days of ICU admission.

### Follow-up Data Collection

Follow-up data collection is described in Supplemental Table 1.

### Feasibility Data Collection

Feasibility was assessed through (1) participant enrollment, (2) data capture rates, and (3) follow-up rates at 3 and 12 months after ICU discharge. Based on our existing population of potential candidates in the Kingston Health Sciences Centre (KHSC) ICU, feasibility of enrollment was defined as a rate of 1 patient per month. Feasibility of data capture was defined as a data capture rate of 80%, in accordance with the literature on similar existing studies.^9^ Feasibility for long-term follow-up data collection was defined as a rate of 70% of ICU survivors, as refusal rates for participation in follow-up research among this group are known to be high, with over one third of participants refusing participation. Reasons for missing data were determined to optimize data collection in the future larger study.

## Results

### Participant Enrollment and Clinical Data

**Figure 1.**
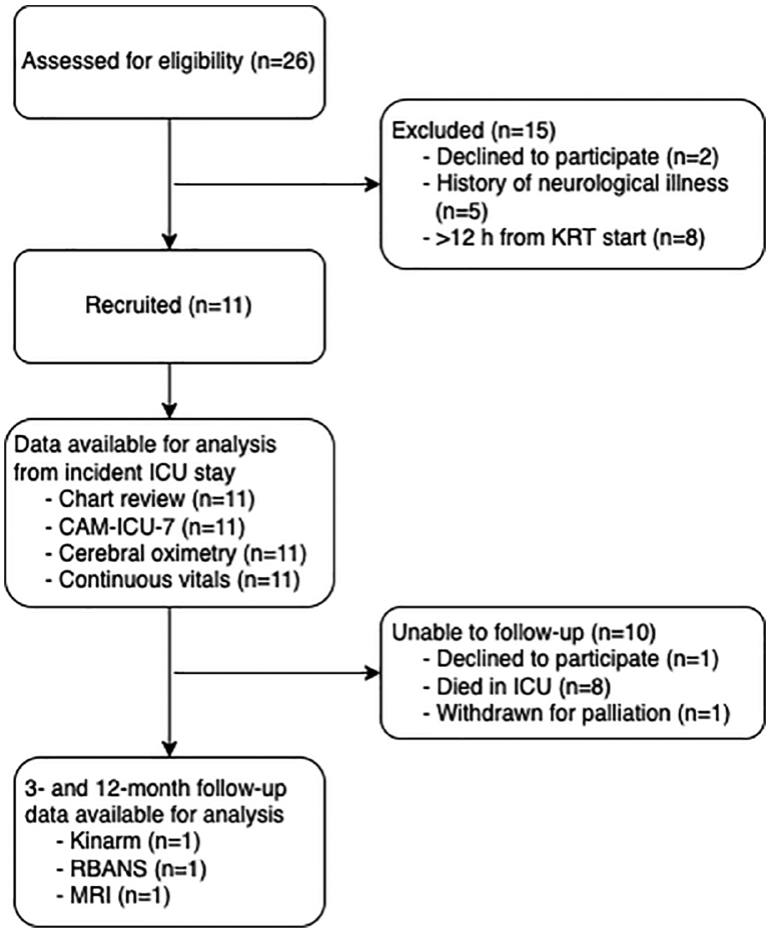
Participant enrollment. *Note.* KRT = kidney replacement therapy; ICU = intensive care unit; CAM = Confusion Assessment Method; RBANS = Repeatable Battery for the Assessment of Neuropsychological Status; MRI = magnetic resonance imaging.

Participant enrollment is summarized in Figure 1. Clinical and demographic data are outlined in Supplemental Table 2. Seven of 11 patients met the CAM screening criteria for delirium during their ICU admission. The mean length of time that patients were delirious was 4.57 (SD ± 4.12) days.

### Data Capture Rates

The baseline data capture rate, including CDR, CFS, and APACHE II, was 100%. For all 11 patients we recorded continuous vitals and cerebral oximetry with a mean capture rate of 91.3%. Missing data was primarily due to the near-infrared spectroscopy sensor being removed for surgical interventions, procedures, or imaging that was not compatible with the oximeter. Data illustrating the feasibility of collection of continuous vitals and cerebral oximetry during participants’ admissions are shown in Supplemental Figure 1. Daily CAM-ICU-7 delirium screening and chart review were performed for all patients with 100% data capture rate.

### Follow-up Rates

Three- and 12-month follow-up testing was completed on only 1 of 3 patients who survived to ICU discharge and included structural T1- and diffusion-weighted brain MRI, Repeatable Battery for the Assessment of Neuropsychological Status (RBANS), and Kinarm assessments. Reasons for lack of follow-up are described in Figure 1. Overall follow-up rate was 50% for patients who were alive at the time of follow-up at 3 and 12 months (ie, 1 out of 2 patients; the third patient died within 1 month of discharge).

## Discussion

Our work demonstrates the feasibility of this study for the primary aim of examining the relationship between intradialytic rSO_2_ and ICU delirium. However, we have identified barriers and resource planning issues that need to be considered for our larger study. The high mortality rate in our study cohort (8/11 patients in ICU, 1/11 patients within 1 month of discharge) is a notable barrier to long-term follow-up. Importantly, performing this early assessment of feasibility has allowed us to identify strategies to ensure that we can obtain sufficient sample sizes, follow-up rates, and data quality to address our outcomes of interest.

Our enrollment rate was 0.61 patients per month. As this was less than our expected 1 per month, strategies to increase our enrollment will need to be implemented to make a larger study feasible. One of the major barriers to participant enrollment in our feasibility study was the inability to enroll patients within the 12-hour window from their time of KRT initiation. This was primarily due to the inability to reach substitute decision-makers if patients initiated KRT overnight, as well as late notification of new eligible patients to the study team. As we begin our larger study, we plan to implement a deferred consent model, with well-described written study materials for participants/proxies to aid in the conversation to notify patients of their enrollment. To address the issue of lack of awareness of eligible patients admitted after hours, we plan to more closely integrate charge nurses into the enrollment process, to ensure that our study team is notified early on of eligible patients. Due to the COVID-19 pandemic, increased ICU admission rates created the need for subsidiary ICU beds that did not have the ability to perform continuous vitals monitoring, thus excluding potentially eligible patients. Due to the decreasing number of COVID-19-related ICU admissions, we expect this issue to diminish over time. Furthermore, our center is currently implementing bedside monitors that can continuously capture data throughout all beds in the hospital, which will permanently resolve this problem.

As our expected follow-up rate was 70%, our follow-up data collection in its current form was determined not to be feasible. Longitudinal research in this population is challenging due to high mortality rates both in ICU and following discharge, high study withdrawal rates, and loss to follow-up. Participants’ poor physical, financial, emotional, psychiatric, and neurocognitive states all contribute to their inability or unwillingness to return for assessment. To reduce attrition, our larger INCOGNITO-AKI study will employ a multifaceted approach, including pairing clinical and research visits, enhancing feasibility of participant attendance at appointments and visit completion, increasing study visibility through regular telephone communications, building rapport between participants and study teams, and employing multiple methods for contacting participants. When employing a combination of these strategies, studies have achieved follow-up rates of 70% to 80%.^10^ Furthermore, we plan to onboard additional centers with the infrastructure available to support this study across Canada, which will enable us to achieve the sample size required to adequately answer our research aims.

## Supplemental Material

sj-docx-1-cjk-10.1177_20543581231192743 – Supplemental material for Neurological Impairment in Critically Ill Patients on Dialysis: Research Letter for the INCOGNITO-AKI Feasibility Study Click here for additional data file. Supplemental material, sj-docx-1-cjk-10.1177_20543581231192743 for Neurological Impairment in Critically Ill Patients on Dialysis: Research Letter for the INCOGNITO-AKI Feasibility Study by Natasha A. Jawa, Samuel A. Silver, Rachel M. Holden, Stephen H. Scott, Andrew G. Day, Patrick A. Norman, Benjamin Y. M. Kwan, David M. Maslove, John Muscedere and J. Gordon Boyd in Canadian Journal of Kidney Health and Disease
