# Supplementary material for: Neurological Impairment in Critically Ill Patients on Dialysis: Research Letter for the INCOGNITO-AKI Feasibility Study
Source: Can J Kidney Health Dis. 2023 Aug 24;10:20543581231192743. doi: 10.1177/20543581231192743 (PMC10461308; doi:10.1177/20543581231192743)
Supplement: sj-docx-1-cjk-10.1177_20543581231192743 – Supplemental material for Neurological Impairment in Critically Ill Patients on Dialysis: Research Letter for the INCOGNITO-AKI Feasibility Study [file sj-docx-1-cjk-10.1177_20543581231192743.docx]

**SUPPLEMENTAL FIGURES & TABLES**

**Supplemental Table 1:** Follow-up measurements.

| **Measure** | **Description** | **Timepoints** |
| --- | --- | --- |
| **RBANS** | Repeatable Battery for the Assessment of Neuropsychological Status. The RBANS is a 30-minute tool to screen and quantify cognitive impairment across a variety of domains among adults, including visuospatial/constructional function, language, attention, and immediate and delayed memory. | 3 months  12 months |
| **Kinarm** | The Kinarm End-Point Lab (Kinarm, Kingston, Ontario, Canada) quantifies neurocognitive impairment in the domains of perceptual motor, complex attention, learning, memory, and executive function. | 3 months  12 months |
| **MRI** | Magnetic resonance imaging. Brain volumetry, fractional anisotropy, and mean diffusivity was measured on MRI. | 3 months  12 months |
| **MARS** | Medication Adherence Rating Scale. To measure adherence to patient medications. | 12 months |
| **MDBQ** | Manchester Driver Behavior Questionnaire. For assessing driving safety. | 12 months |
| **Adverse events** | Adverse events are defined as hospitalizations and emergency department visits. | 12 months |

**Supplemental Table 2:** Clinical and demographic data.

| Demographic | Units | Participants (n=11) | |
| --- | --- | --- | --- |
| Age | mean, SD | 67.5 | 12.7 |
| Sex, male | n (%) | 7 | 63.6 |
| Days on CKRT | mean, SD | 7.5 | 9.2 |
| Days on IHD | mean, SD | 1.8 | 2.7 |
| eGFR at KRT start (mL/min/1.73m^2^) | mean, SD | 15.0 | 8.8 |
| Serum creatinine (µmol/L) | mean, SD | 373.8 | 131.3 |
| ICU length of stay | mean, SD | 19.9 | 33.2 |
| Mechanical ventilation | n (%) | 1 | 9.1 |
| Admitting diagnosis | n (%) | 1 | 9.1 |
| Endocarditis |  | 1 | 9.1 |
| Acute kidney injury |  | 1 | 9.1 |
| Sepsis |  | 1 | 9.1 |
| Necrotizing fasciitis |  | 1 | 9.1 |
| Cellulitis |  | 1 | 9.1 |
| Acute coronary syndrome |  | 1 | 9.1 |
| Aortic stenosis |  | 1 | 9.1 |
| Pneumonia |  | 2 | 18.2 |
| Post-coronary artery bypass graft |  | 1 | 9.1 |
| Perforated bowel |  | 1 | 9.1 |

**Supplemental Figure 1:** Continuous vitals and cerebral oximetry data capture. **
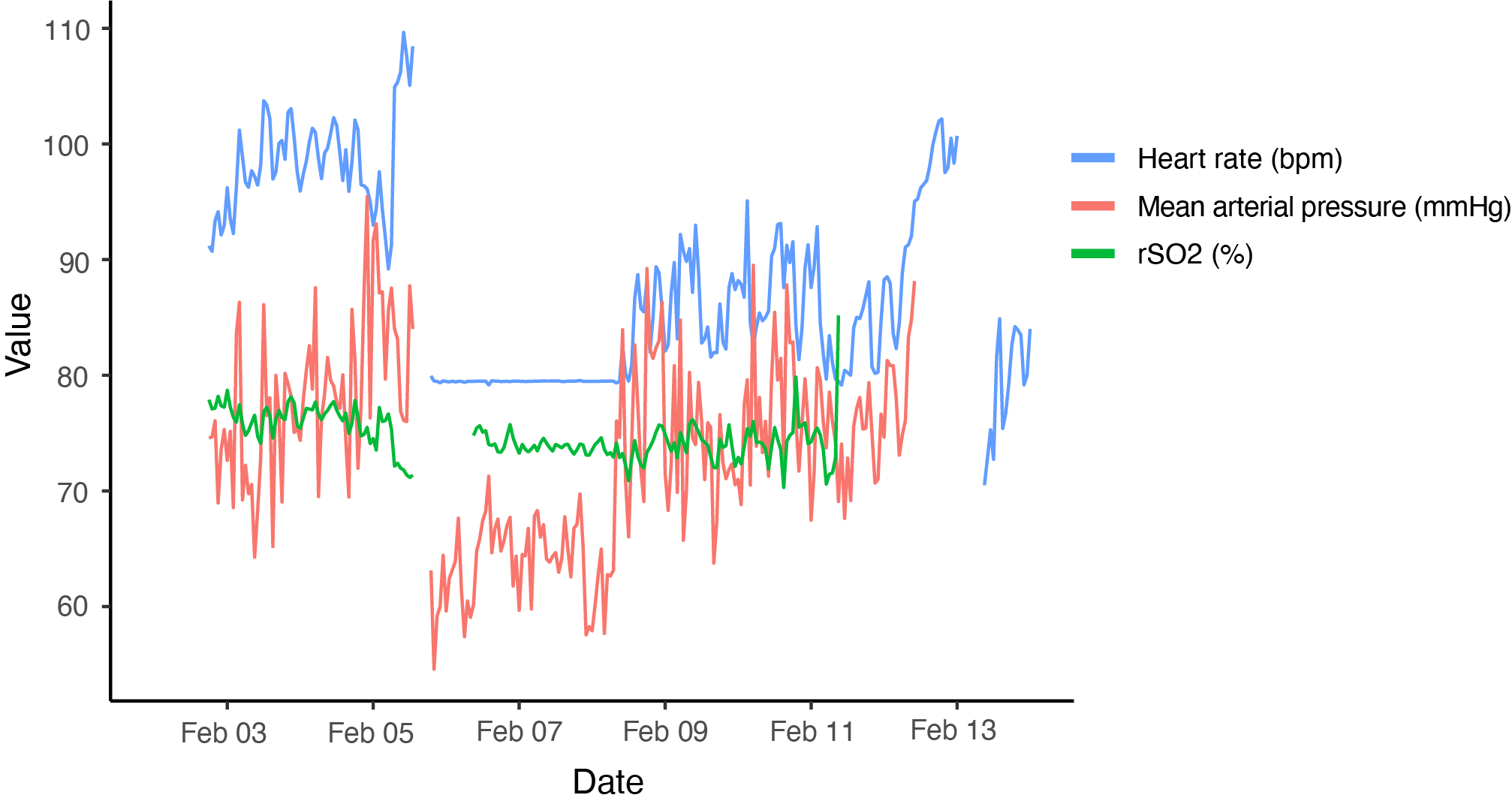
**

**
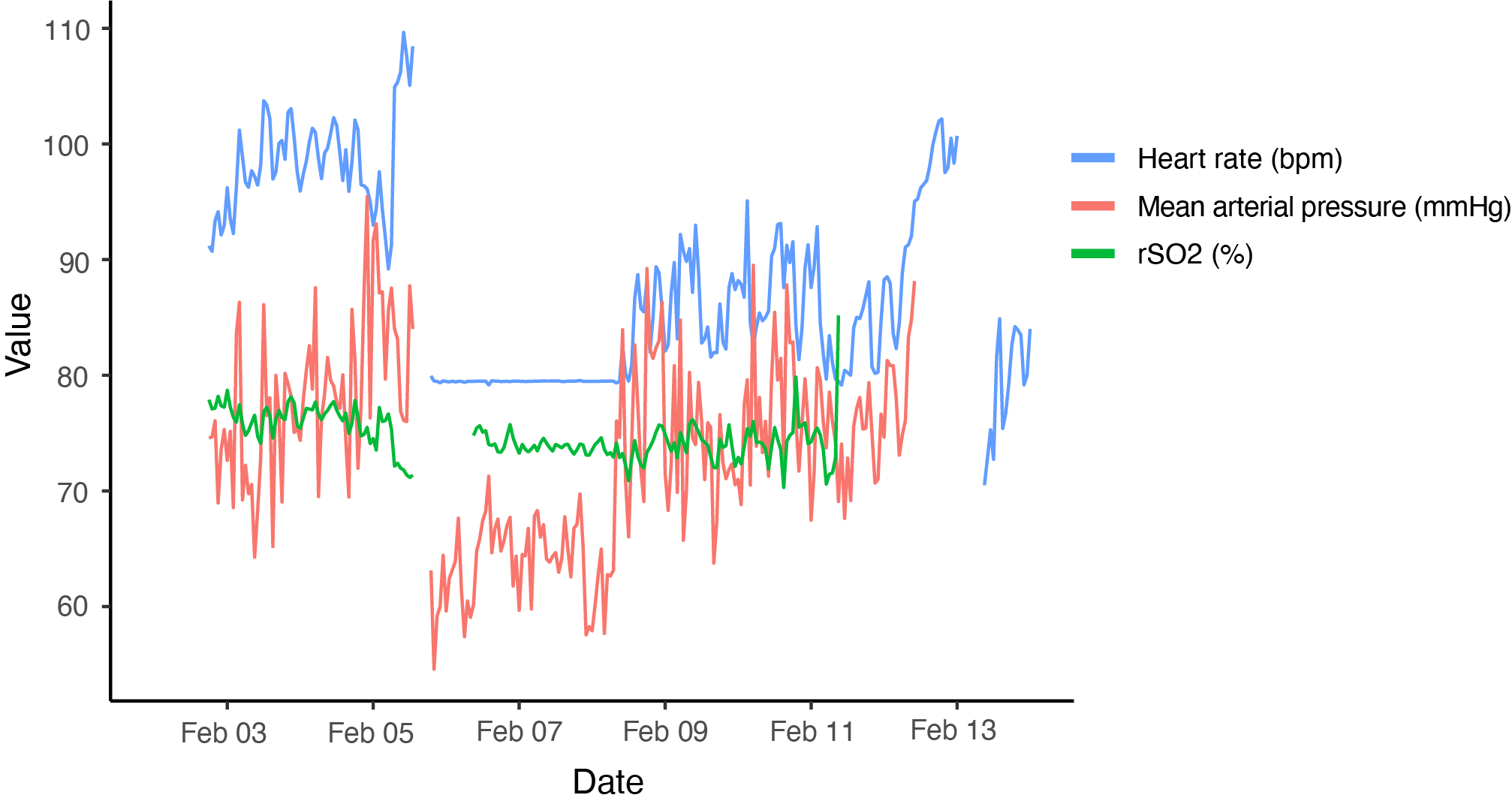
**

Heart rate, mean arterial pressure, and continuous rSO2 data for the first enrolled patient. Patient was on CKRT from February 2, 2022 to February 8, 2022 and subsequently transitioned to IHD on February 11, 2022 and February 13, 2022.
